# A comparison between asymptomatic and symptomatic ureteral stones

**DOI:** 10.1038/s41598-023-29866-5

**Published:** 2023-02-16

**Authors:** Tae Il Noh, Jong Hyun Pyun, Ji Sung Shim, Seok Ho Kang, Jun Cheon, Sung Gu Kang

**Affiliations:** 1 Department of Urology, Anam Hospital, Korea University College of Medicine, 73, Goryeodae-Ro, Seongbuk-Gu, Seoul, 02841 Korea; 2 Department of Urology, Sungkyunkwan University School of Medicine, Seoul, Republic of Korea

**Keywords:** Urology, Ureter

## Abstract

To investigate the characteristics and impact of asymptomatic (silent) ureteral stones on renal function and compare them with those of symptomatic stones. We retrospectively reviewed the medical records of 677 patients who underwent ureteroscopic lithotripsy or laparoscopic ureterolithotomy for ureteral stones between 2016 and 2020. Patients were divided into two groups according to the presence of recognizable symptoms. We investigated the characteristics and impact of silent stones on post-treatment renal function recovery and compared them with those of symptomatic stones. Among the 677 patients, 43 (6.4%) had asymptomatic ureteral stones, and 634 (93.6%) had symptomatic ureteral stones. Compared to symptomatic stones, asymptomatic stones were larger (11.4 mm vs. 9.6 mm, *p* = 0.003), more commonly present in the upper ureter (62.7% vs. 48.0%, *p* = 0.04), and more commonly associated with high-grade hydronephrosis (32.8% vs. 12.3%, *p* < 0.001); however, no difference in metabolite composition was observed between the two group of stone. In the asymptomatic stone group, the mean preoperative estimated glomerular filtration rate (eGFR) was 77.37 ± 23.54 mL/min/1.73 m^2^, and the mean postoperative eGFR indicated no significant improvement at 1 day, 7 days, 3 months, and 12 months (76.66 ± 21.45, 77.89 ± 20.87, 77.29 ± 22.22, and 76.71 ± 24.21 mL/min/1.73 m^2^, respectively; *p* = 0.567, *p* = 0.613, *p* = 0.924, and *p* = 0.202, respectively). In the symptomatic stone group, the mean preoperative eGFR was 78.17 ± 28.25 mL/min/1.73 m^2^; the mean postoperative eGFRs at 1 day, 7 days, 3 months, and 12 months were 81.24 ± 26.38, 86.16 ± 25.61, 89.11 ± 25.43, and 89.50 ± 26.01 mL/min/1.73 m^2^, respectively and demonstrated significant improvement (*p* = 0.002, *p* < 0.001, *p* < 0.001, and *p* < 0.001, respectively). Silent stones irreversibly impaired renal function, even after proper management. Therefore, active treatment strategies are required for all patients who are hesitant to receive treatment for silent stones because of their asymptomatic status to prevent permanent renal impairment.

## Introduction

Ureterolithiasis is one of the most common urologic diseases worldwide, and the prevalence of stone disease has increased steadily due to changes in diet and lifestyle^1–3^. Ureteral stones usually present with acute symptoms because of urinary tract obstruction. Possible symptoms of urinary calculi include acute or chronic flank pain, hematuria, and symptoms related to urinary tract infection such as dysuria, frequency, and fever^4^. Additionally, without proper treatment, obstructive uropathy caused by calculi can occasionally lead to permanent renal dysfunction^5^.

Asymptomatic (silent) stones in the renal collecting system are diagnosed frequently by ultrasonography and abdominal non-contrast computed tomography (CT) during regular health check-ups. Their impact and natural course have been thoroughly discussed^6,7^. Owing to the development of endoscopic equipment, diagnosed asymptomatic stones in the collecting system have been treated relatively easily; however, studies on the impact and natural course of asymptomatic (silent) stones in the ureter have been overlooked and neglected^8,9^. Although several studies focusing on the influence of silent stones on renal function have been reported, studies comparing silent and symptomatic ureteral stones are rare^10^. In this study, we aimed to investigate the characteristics and impact of silent stones on renal function recovery after proper treatment by comparing the cases of asymptomatic and symptomatic ureteral stones, which were treated during the same period and single center.

## Materials and methods

### Study design

**Figure 1:**
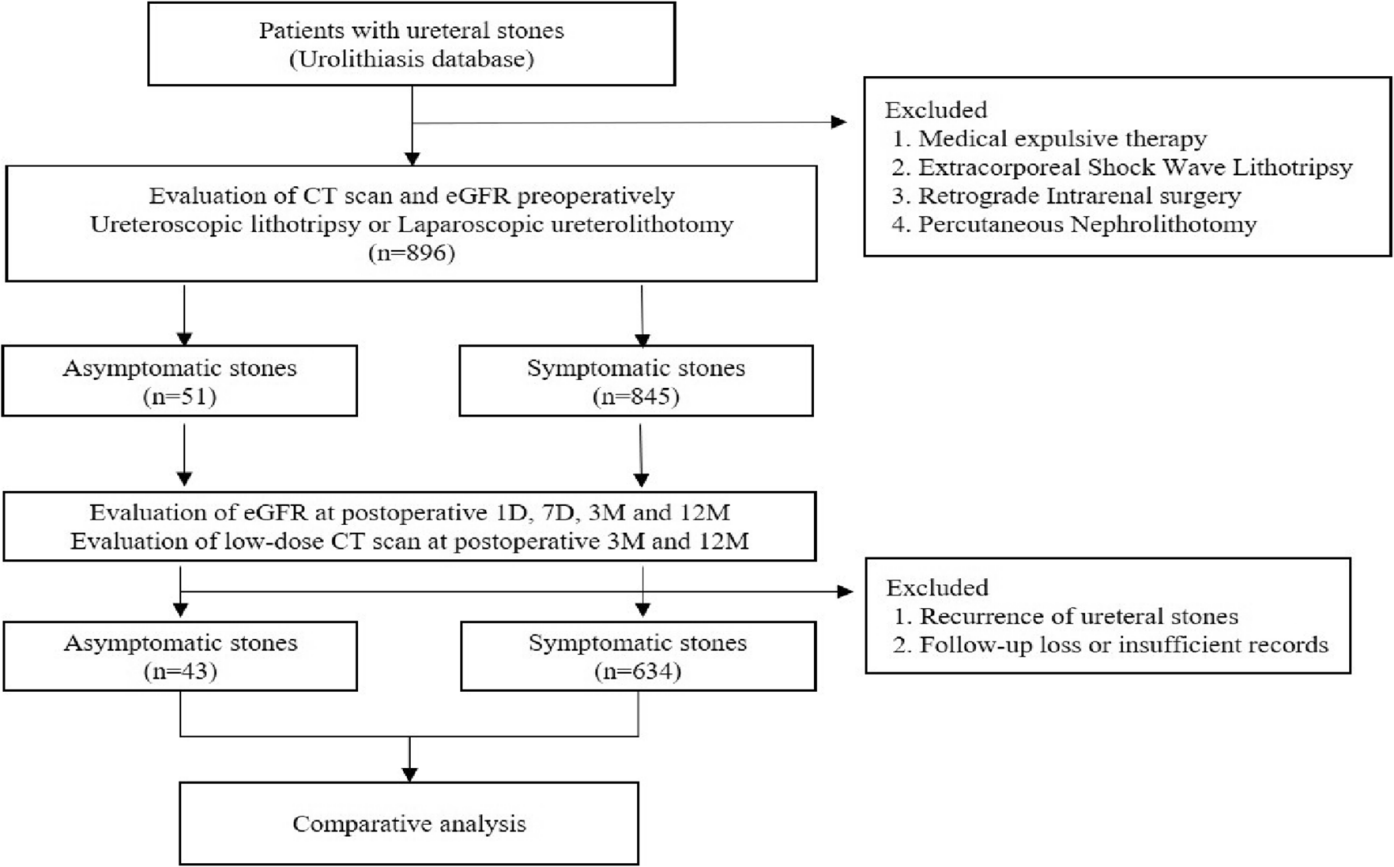
Flowchart for study design. CT, computed tomography; D, day; eGFR, estimated glomerular filtration rate; M, month.

We retrospectively reviewed the medical records of 677 patients who underwent endoscopic surgery such as ureteroscopic lithotripsy and laparoscopic ureterolithotomy for ureteral stones between 2016 and 2020. Patients who underwent medical expulsive therapy (MET), extracorporeal shock wave lithotripsy, percutaneous nephrolithotomy, or retrograde intrarenal surgery were excluded from the study in order to assess the course of renal function recovery without any interference from the associated effects of kidney stones or surgical methods. According to the presence of recognizable symptoms such as pain, gross hematuria, and urinary tract infection, patients were divided into two groups: symptomatic and asymptomatic stone groups. In addition, we included that there were no symptoms at the time of diagnosis. Although initial stone related symptoms were reported, they were ignored; hence, we did not find any symptoms at the timing of diagnosis. All the patients in the symptomatic stone group received surgical treatment because of persistent pain or when the spontaneous passage of stones was unlikely to occur over 3 weeks, particularly ureteroscopic lithotripsy for stones > 6 mm and laparoscopic ureterolithotomy for stones > 20 mm^11^. Likewise, patients with silent ureteral stones underwent surgical treatment with the same indications for symptomatic stones except the stone-related symptoms. We compared the findings of metabolic analysis and urinary analysis, the size and location of stone, and the grade of hydronephrosis evaluated by CT scan between the two groups. To investigate renal function recovery, we evaluated the differences between the preoperative estimated glomerular filtration rate (eGFR) and 1-day, 7-day, 3-month, and 12-month postoperative values of eGFR and we compared these differences between the two groups (Fig. 1).

The eGFR values were calculated based on the Chronic Kidney Disease Epidemiology Collaboration (CKD-EPI) equation using serum creatinine levels as follows: 141 × min(SCr/κ,1)^α^ × max(SCr/κ,1) ^−1.209^ × 0.993^age^ × 1.018 [if female] × 1.159 [if African American], where κ is 0.7 for female patients and 0.9 for male patients; α is − 0.329 for female patients and  − 0.411 for male patients; min indicates the minimum of SCr/κ or 1 and max indicates the maximum of SCr/κ or 1. To monitor the change in hydronephrosis, we performed serial low-dose CT scan at 3 months and 12 months postoperatively.

### Statistical analysis

Demographic and clinical variables were summarized using descriptive statistics. Continuous variables are presented as mean (standard deviation). Independent and paired t-tests were performed to compare mean values between the two groups. The Shapiro–Wilk test was used to assess the assumption of normality. Fisher’s exact test and a chi-square test were used for analyzing categorical variables, which are presented as frequency (percentage). Logistic regression was used to identify the predisposing factors for asymptomatic ureteral stones. All statistical analyses were performed using SPSS version 26.0, and a p-value of < 0.05 was considered statistically significant.

### Ethics approval

This study was conducted in accordance with the Declaration of Helsinki and current ethical guidelines. The study was reviewed and approved by the Ethics Committee and Institutional Review Board of Korea University Anam Hospital (IRB No. 2020AN0189). Given the retrospective nature of this study, the need to obtain informed consent has been waived by the Ethics Committee and Institutional Review Board of Korea University Anam Hospital.

## Results

Among 677 patients, 43 (6.4%) had silent ureteral stones and the remaining 634 (93.6%) had symptomatic ureteral stones. In 72.1% (31/43) cases, silent stones were diagnosed via ultrasonography (48.8%) or microscopic hematuria (23.3%) during regular heath examinations, whereas 27.9% of patients were diagnosed with asymptomatic stones during work-up studies for other diseases (e.g. pancreatic cancer, gastric cancer) (Table 1).

**Table 1:** Incidental diagnosis of silent stones.- ‘Health screening’ and ‘Other disease work-up’ are the same heading line, and ‘Cancer work-up’ and ‘Lymphoma’ are subcategories of ‘Other disease work-up’.

Detection	Asymptomatic stones (n = 43)
Health screening	
Ultrasonography (%)	21 (48.8)
Microscopic hematuria (%)	10 (23.3)
Other disease work-up (%) Cancer work-up Lymphoma	12 (27.9) 11 (25.6) 1 (2.3)

Patients in the symptomatic ureteral stone group had flank or abdominal pain with or without gross hematuria and urinary tract infection. All patients underwent laparoscopic ureterolithotomy or ureteroscopic lithotripsy using the holmium laser. Of the 43 patients with asymptomatic stones, 5 (81.4%) underwent ureteroscopic lithotripsy, and 8 (18.6%) underwent laparoscopic ureterolithotomy. Among the 634 patients with symptomatic stones, 608 (95.9%) underwent ureteroscopic lithotripsy, and 26 (4.1%) underwent laparoscopic ureterolithotomy. There were no significant differences in mean age (57.6 years vs. 55.5 years), the proportion of men (65.1% vs. 60.6%), body mass index (24.9 kg/m^2^ vs. 24.6 kg/m^2^), previous history of stone disease (37.2% vs. 36.0%), and underlying disease, such as hyperlipidemia (20.9% vs. 19.6%), hypertension (44.1% vs. 44.5%), and chronic kidney disease (6.9% vs. 4.1%; all *p* > 0.05) between the asymptomatic stone group and symptomatic stone group. However, the proportion of patients with a history of diabetes mellitus was significantly higher in the asymptomatic stone group than in the symptomatic stone group (27.9% vs. 19.6%, *p* < 0.001) (Table 2).

**Table 2:** Patient characteristics.

Characteristic	Asymptomatic stones	Symptomatic stones	*p*-value
Number of patients (%)	43 (6.4)	634 (93.6)	
Age (year), mean ± SD	57.6 ± 11.5	55.5 ± 13.6	0.162^b^
Sex			0.102^d^
Male (%)	28 (65.1)	384 (60.6)	
Female (%)	15 (34.9)	252 (39.4)	
Body mass index (kg/m^2^), mean ± SD	24.9 ± 3.0	24.6 ± 3.2	0.504^b^
Underlying disease (%)			
DM	12 (27.9)	30 (4.7)	< 0.001^c^
Hyperlipidemia	9 (20.9)	122 (19.6)	0.769^d^
HTN	19 (44.1)	282 (44.5)	0.837^d^
CKD	3 (6.9)	26 (4.1)	0.241^d^
Previous history of stone	16 (37.2)	228 (36)	0.979^c^
Surgical method			
Ureteroscopic ureterolithotripsy	35 (81.4)	608 (95.9)	
Laparoscopic ureterolithotomy	8 (18.6)	26 (4.1)	
Hydronephrosis grade (%)			< 0.001^a^
0	3 (6.9)	34 (5.4)	
I	17 (39.2)	350 (55.2)	
II	9 (21.1)	172 (27.1)	
III	3 (7.2)	56 (8.8)	
IV	11 (25.6)	22 (3.5)	
Location			0.04^a^
Upper ureter	27 (62.7)	304 (48.0)	
Mid ureter	6 (14.0)	64 (10.1)	
Lower ureter	6 (14.0)	222 (35.0)	
Multiple locations in the ureter	4 (9.3)	44 (6.9)	
Lateralization			0.007^a^
Left	15 (34.9)	328 (51.7)	
Right	26 (60.5)	274 (43.2)	
Bilateral	2 (4.6)	32 (5.0)	
Stone size (mm), mean ± SD	11.4 ± 4.6	9.6 ± 4.6	0.003^b^
Stone count, mean ± SD	1.3 ± 0.6	1.2 ± 0.6	0.611^b^
Urine analysis (pH)	5.8 ± 0.8	6.0 ± 0.9	0.060^b^

*SD* standard deviation, *DM* diabetes mellitus, *HTN* hypertension, *CKD* chronic kidney disease.

^a^Chi-square test.

^b^Independent *t*-test.

^c^Chi-square test.

^d^Fisher’s exact test.

### Stone characteristics

Table 2 shows the characteristics of ureteral stones in both the groups. There were significant differences in the distribution of hydronephrosis grade, stone location, stone lateralization, and mean stone size (all *p* < 0.05) between the two groups. In the asymptomatic stone group, hydronephrosis was observed in 40 patients (93.1%). Among them, 17 (39.2%), 9 (21.1%), 3 (7.2%), and 11 (25.6%) patients showed grade I, grade II, grade III, and grade IV hydronephrosis, respectively. Of 600 patients (94.6%) with hydronephrosis in the symptomatic stone group, 350 (55.2%) had grade I, 172 (27.1%) had grade II, 54 (8.8%) had grade III, and 22 (3.5%) had grade IV hydronephrosis. The proportion of patients with high grade hydronephrosis was higher in the asymptomatic stone group (*p* < 0.001). In the asymptomatic stone group, stones were found in the upper ureter in 27 (62.7%) patients; mid ureter, 6 (14.0%) patients; lower ureter, 6 (14.0%) patients; and multiple regions of the ureter, 2 (4.6%) patients. Regarding the location of silent stones, 63% of stones were located in the right ureter, while 34.6% of stones were located in the left ureter; only 2.5% of stones occurred bilaterally. Symptomatic stones were found in the upper ureter in 304 (48.0%) patients; mid ureter, 64 (10.1%) patients; lower ureter, 222 (35.0%) patients; and multiple regions of the ureter, 44 (6.9%) patients. Regarding the location of symptomatic stones, 51.7% of stones were located on the left side, while 43.2% of stones were located on the right side; only 5% of stones occurred bilaterally. In the asymptomatic stone group, the proportion of upper ureteral stones was significantly higher than that in symptomatic stone group (*p* < 0.001).

Mean stone size in the asymptomatic stone group was greater than that in the symptomatic stone group (11.4 mm vs. 9.6 mm, *p* = 0.003). However, there were no significant differences in mean stone count (1.3 vs. 1.2, *p* = 0.611) and urine pH (5.8 vs. 6.0, *p* = 0.060) between the two groups.

According to stone-specific metabolic evaluation (Table 3), the most common type of stone (39.5% of asymptomatic stones and 37.9% of symptomatic stones) was found to be composed of a mixture of calcium, oxalate, and phosphate, while the proportion of uric acid stones (11.6% and 8.1%) was the lowest in both the groups. There was no difference in the distribution of metabolic type of stones (*p* > 0.05).

**Table 3:** Metabolic characteristics of stones.

	Asymptomatic stones	Symptomatic stones	*p*-value*
Metabolic characteristics (%)			0.684
Calcium phosphate	10 (23.3)	210 (33.3)	
Calcium oxalate	11 (25.6)	134 (20.7)	
Calcium oxalate + calcium phosphate	17 (39.5)	240 (37.9)	
Uric acid	5 (11.6)	50 (8.1)	

*Fisher’s exact test.

### Kidney function

**Figure 2:**
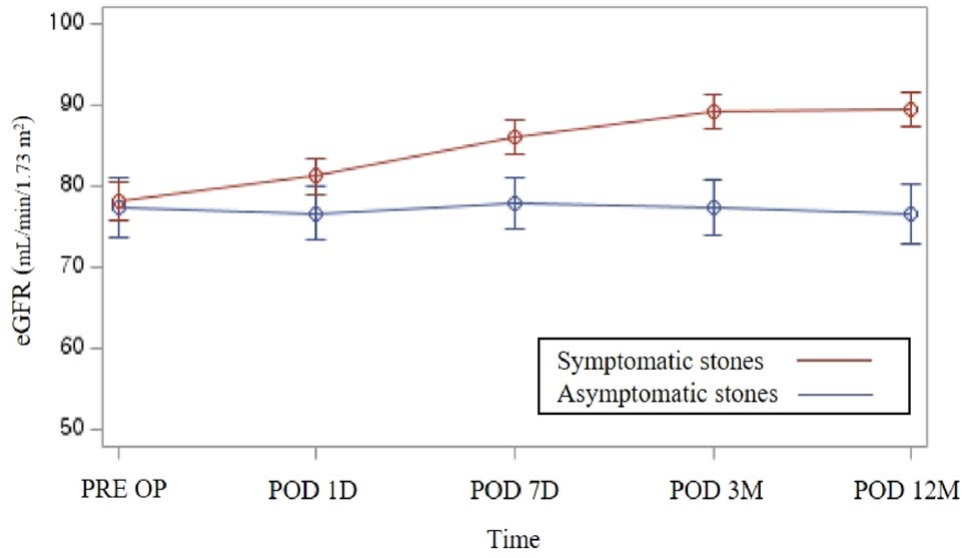
Comparing the impact on eGFR between asymptomatic and symptomatic ureteral stones.

Table 4 presents the impact of ureteral stones on postoperative renal function recovery with changes in eGFR in each group, which were normally distributed at a significance level of 0.05. In the asymptomatic stone group, the mean preoperative eGFR was 77.37 ± 23.54 mL/min/1.73 m^2^. No significant improvement in mean eGFR at 1 day (76.66 ± 21.45 mL/min/1.73 m^2^, *p* = 0.567), 7 days (77.89 ± 20.87 mL/min/1.73 m^2^, *p* = 0.613), 3 months (77.29 ± 22.22 mL/min/1.73 m^2^, *p* = 0.924), and 12 months (76.71 ± 24.21 mL/min/1.73 m^2^, *p* = 0.202) after treatment was observed in the asymptomatic stone group. In the symptomatic stone group, the mean preoperative eGFR was 78.17 ± 28.25 mL/min/1.73 m^2^. The preoperative eGFR indicated no significant difference between the two groups (*p* = 0.865). Conversely, significant gradual improvement was observed in the mean eGFR at 1 day (81.24 ± 26.38 mL/min/1.73 m^2^, *p* = 0.002), 7 days (86.16 ± 25.61 mL/min/1.73 m^2^, *p* < 0.001), 3 months (89.11 ± 25.43 mL/min/1.73 m^2^, *p* < 0.001), and 12 months (89.50 ± 26.01 mL/min/1.73 m^2^, *p* < 0.001) postoperatively in the symptomatic stone group. The postoperative eGFR indicated a significant difference from postoperative day 7 (Fig. 2).

**Table 4:** Impact of asymptomatic and symptomatic stones on postoperative renal function.

eGFR value	Asymptomatic stones	*p*-value*	Symptomatic stones	*p*-value*	*p*-value**
PRE OP	77.37 ± 23.54		78.17 ± 28.25		0.865
POD 1D	76.66 ± 21.45	0.567	81.24 ± 26.38	0.002	0.299
POD 7D	77.89 ± 20.87	0.613	86.16 ± 25.61	< 0.001	0. 055
POD 3 M	77.29 ± 22.22	0.924	89.11 ± 25.43	< 0.001	0.007
POD 12 M	76.71 ± 24.21	0.202	89.50 ± 26.01	< 0.001	0.005

*eGFR* estimated glomerular filtration rate, *PREOP* preoperative, *POD* postoperative day, *D* day, *M* month.

*The mean postoperative eGFR value was compared with the mean preoperative eGFR value using a paired *t*-test.

**The mean eGFR value of symptomatic stones was compared to the mean eGFR value of asymptomatic stones using an independent *t*-test.

### Changes in hydronephrosis grade

**Figure 3:**
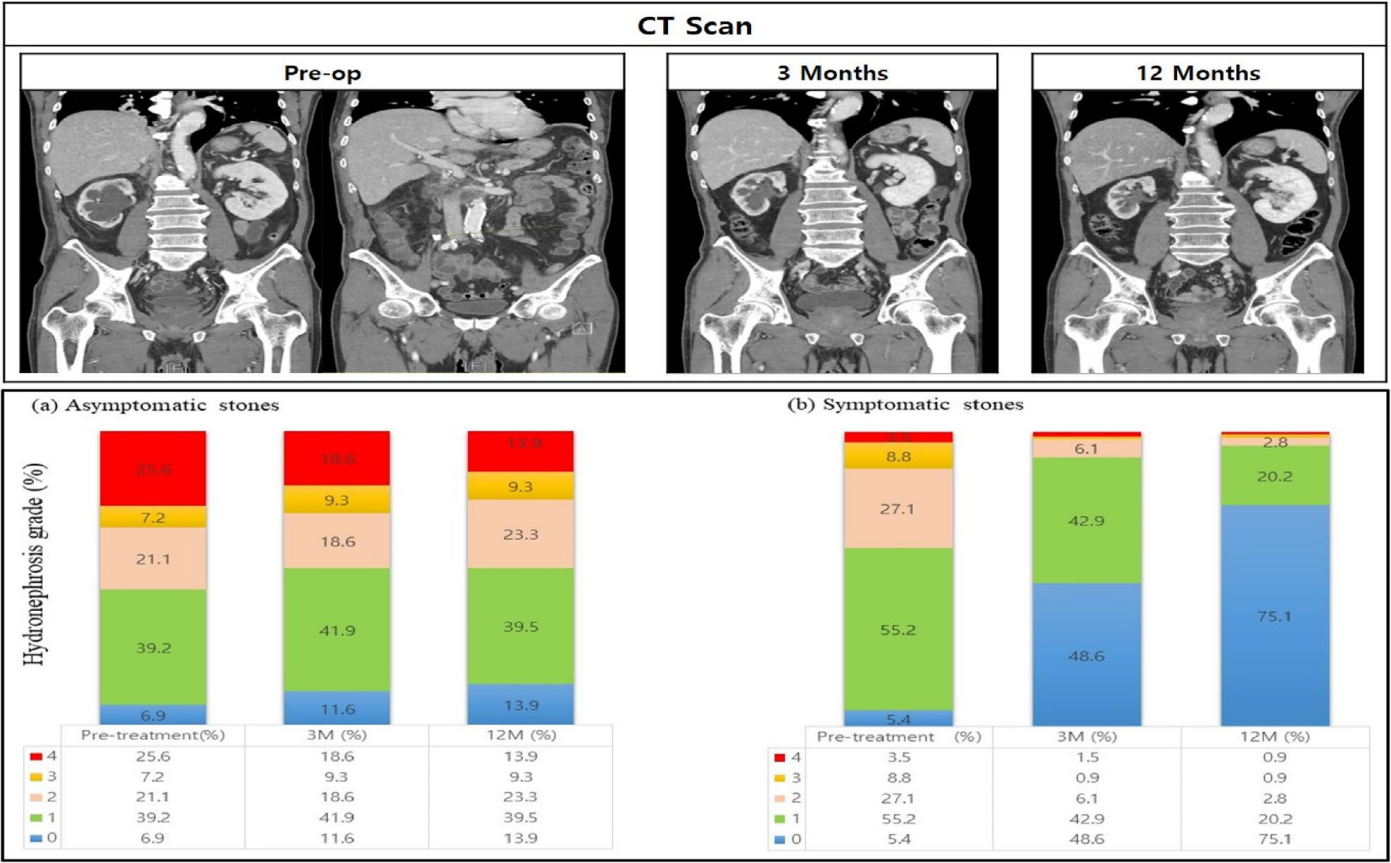
Changes in hydronephrosis grade estimated by serial computed tomography after stone treatment.

High-grade hydronephrosis (grade III) was more common in the asymptomatic stone group than in the symptomatic stone group (32.8% vs. 12.3%, *p* < 0.001). In the symptomatic stone group, serial CT showed significant improvement in hydronephrosis grade 3 months after surgical management of stones; the proportion of patients with no hydronephrosis/grade I hydronephrosis was 95.3% at 12 months post-treatment. The proportions of patients with high-grade hydronephrosis in the asymptomatic stone group were 27.9% and 23.2% at 3 months and 12 months post-treatment. The proportion of patients with each hydronephrosis grade did not change significantly in the asymptomatic stone group (*p* = 0.113) (Fig. 3).

## Discussion

The natural history of symptomatic ureteral calculi has been intensively studied, and the representative symptom of ureteral stones is renal colic caused by partial or complete obstruction of the ureter^12,13^. Renal colic is thought to arise from the stretching and spasm of smooth muscles in the wall of the renal pelvis and ureter, with increases in renal pelvic pressure and blood flow and stimulation of nerve endings^14,15^. Silent stones were defined as stones that present no subjective stone-related symptoms such as acute or chronic flank pain, gross hematuria, or urinary tract infection. In this study, silent stones were found in 6.4% of all patients who were treated surgically in the same period and single center. The proportion of silent stones was reported in the range of 1.1–5.3%^16–18^. Furthermore, in a large cohort study of adults with asymptomatic stones, the real prevalence of silent stone was 7.8% (395 in 5047)^19^. In this study, we included the patients who were converted from patients with symptomatic stones to patients with asymptomatic stones; they ignored initial mild symptoms, and at the timing of diagnosis, symptoms were relieved. Although several studies were conducted to investigate silent stones, the characteristics and impact of silent stones on renal function recovery are not clear due to the small number of cases included in those studies. Furthermore, the converted silent stone patients would not have a chance to receive treatment unless the stones were incidentally diagnosed during regular check-up or clinical evaluation for the other diseases. Therefore, these patients were important for investigating the characteristics and impact of silent stones.

In this comparative study, silent stones were larger (11.4 mm vs. 9.6 mm, *p* = 0.003) and more frequently present in the upper ureter (62.7% vs. 48.0%, *p* = 0.04) than symptomatic stones. The proportion of patients with high-grade hydronephrosis in the asymptomatic stone group was higher than that in the symptomatic stone group (53.6% vs. 39.4%, *p* < 0.001). Unlike acute obstruction causing a rapid increase in intraluminal pressure and ureteral dilation in the symptomatic stone group, hydronephrosis causes physiological changes and ureteral dilatation with chronic obstruction in the asymptomatic stone group^9^. In symptomatic stone group, it showed significant recovery and improvement of renal function after 7 days postoperatively. Meanwhile, in the asymptomatic stone group, there was no significant difference between preoperative and 7-day to 12-months postoperative mean eGFR, indicating irreversible impairment of renal function. Additionally, there was no significant improvement in high-grade hydronephrosis. The long duration of chronic obstruction with delay in diagnosis and proper treatment may contribute to the irreversible impairment of renal function and persistence of higher-grade hydronephrosis in the asymptomatic stone group^20^.

This study suggested that the cause and origin of silent stones were not significantly different from those of symptomatic stones. To the best of our knowledge, no previous study has compared silent and symptomatic stones and no study has reported a difference in metabolic composition of silent and symptomatic stones though metabolite analysis. The asymptomatic stone group showed the following characteristics: upper ureter location, large stone size, and irreversible impairment of renal function, and persistence of high-grade hydronephrosis even after proper treatment. We evaluated the disease course in each group postoperatively through serial measurements of eGFR and CT follow-up.

Nevertheless, this study has several limitations. The difference in eGFR was calculated based on serum creatinine level, which only measures total renal function, not the function of the affected kidney separately, and does not reflect the compensatory hyper-infiltration in the contralateral kidney. 99mTc-diethylenetriamine pentaacetic acid (99mTc-DTPA) can be used to measure the relative function of the affected kidney^16^. However, only 26 patients were evaluated because of the difficulty to use it routinely in real-world clinical practice and in a large-scale study owing to additional cost and poor patient compliance. Additionally, as this was a retrospective study that excluded patients who received other treatment options, such as MET, ESWL, and observation, confounding factors may have contributed to biased outcomes.

Despite these limitations, we found that the incidence of silent stone was high and silent stones caused irreversible impairment of kidney function, suggesting that the immediate and proper management of the silent stones should not be ignored and overlooked and is essential. Therefore, when we meet patients who are hesitating or ignoring the treatment of silent stones due to no current symptoms, urologists should understand the need of immediate and appropriate treatment of silent stones.

## Conclusion

Silent stone caused irreversible impairment of renal function even after proper management of stones compared to significant improvement and recovery of renal function in the symptomatic stone group. Therefore, active treatment strategies are required for all patients who are hesitant to receive treatment for silent stones because of their asymptomatic status to prevent permanent renal function impairment.

## Data Availability

All data generated or analyzed during this study are included in this article and its supplementary information files. The datasets used and/or analyzed in this study are available from the corresponding author upon reasonable request.
